# 2020 FDA TIDES (Peptides and Oligonucleotides) Harvest

**DOI:** 10.3390/ph14020145

**Published:** 2021-02-11

**Authors:** Othman Al Musaimi, Danah Al Shaer, Fernando Albericio, Beatriz G. de la Torre

**Affiliations:** 1 School of Chemistry and Physics, University of KwaZulu-Natal, Durban 4001, South Africa; musamiau@gmail.com; 2 KRISP, School of Laboratory of Medicine and Medical Science, College of Health Sciences, University of KwaZulu-Natal, Durban 4001, South Africa; danah.shaer@gmail.com; 3 Networking Centre on Bioengineering, Biomaterials and Nanomedicine (CIBER-BBN), Department of Organic Chemistry, University of Barcelona, 08028 Barcelona, Spain; 4 Institute for Advanced Chemistry of Catalonia (IQAC-CSIC), 08034 Barcelona, Spain

**Keywords:** belantamab mafodotin-blmf, ^64^Cu-DOTATATE, drugs, FDA, ^68^Ga-PSMA-11, lumasiran, setmelanotide, oligonucleotides, peptides, viltolarsen

## Abstract

2020 has been an extremely difficult and challenging year as a result of the coronavirus disease 2019 (COVID-19) pandemic and one in which most efforts have been channeled into tackling the global health crisis. The US Food and Drug Administration (FDA) has approved 53 new drug entities, six of which fall in the peptides and oligonucleotides (TIDES) category. The number of authorizations for these kinds of drugs has been similar to that of previous years, thereby reflecting the consolidation of the TIDES market. Here, the TIDES approved in 2020 are analyzed in terms of chemical structure, medical target, mode of action, and adverse effects.

## 1. Introduction

**Figure 1:**
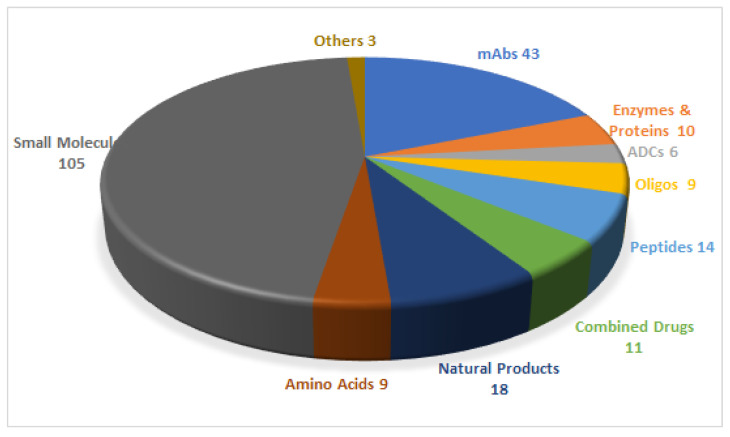
A total of 228 new drugs were approved by the Food and Drug Administration (FDA) from 2016 to 2020 [2]. mAbs, monoclonal antibodies; ADCs, antibody drug conjugates; Oligos, oligonucleotides.

In 2020, the Food and Drug Administration (FDA) has approved 53 drugs [1,2], giving a total of 228 entities (new chemical entities and biologics) authorized during the last 5 years (2016–2020) (Figure 1) [2]. Of the 53 drugs accepted in 2020, six are peptides and oligonucleotides (TIDES). In this regard, the last five years have witnessed the authorization of 23 TIDES. These numbers further strengthen the TIDES market, which, in number of drugs, accounts for around 10% of the total.

Although 2020 can be considered an excellent year from the perspective of both the total number of drugs approved and TIDES, it has been marked by the coronavirus disease 2019 (COVID-19) pandemic. However, this unique situation has allowed confirmation of the robustness of all pharmaceutical industry stakeholders. Indeed, in the space of approximately 10 months, the disease was identified and the first vaccinations were rolled out. From the perspective of TIDES, it is important to highlight that the three first vaccines approved by the regulatory agencies could be considered first-in-class and are based on either RNA or DNA. The explosion of oligonucleotides as drugs in recent years and later as key components of vaccines has been driven by intense research and investment in these intriguing molecules by many academic and industrial groups over more than 30 years.

The four peptides and two oligonucleotides approved by the FDA in 2020 are shown in Table 1, together with the indication, target, and route of administration.

## 2. Oligonucleotides

Oligonucleotides are becoming consolidated as therapeutic drugs after the success of this family in the treatment of various severe genetic disorders, mainly in the past few years. By the end of 2020, a dozen oligonucleotide drugs were on the market, nine of them having received approval since 2016. A summary of all authorized oligonucleotide drugs is shown in Table 2.

### 2.1. Viltolarsen (Viltepso^TM^)

**Figure 2:**
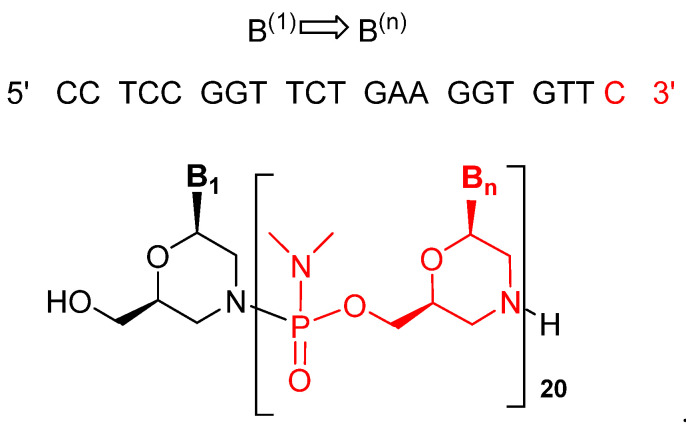
Structure of viltolarsen (Viltepso^TM^).

Viltolarsen is an antisense oligonucleotide consisting of a 21 phosphorodiamidate morpholino oligomer (PMO). In this kind of oligonucleotide analog, the natural sugar-phosphate backbone has been replaced by phosphorodiamidate-morpholino units, leading to a neutral backbone. The sequence of the bases is shown in (Figure 2) [4,5].

This drug is prescribed to treat Duchenne’s muscular dystrophy (DMD) amenable to exon 53 skipping [5]. DMD is an X chromosome-linked genetic disorder in which young patients suffer progressive deterioration of body muscles that ends with death in the second or third decade of life [4]. The dystrophin gene consists of 79 exons that encode for the 427-kDa cytoskeletal protein dystrophin. The protein protects and stabilizes the plasma membrane of muscle cells from the mechanical stress placed on muscle fibers upon contraction [4,6]. The absence of dystrophin damages muscle cell membranes, thereby leading to muscle degeneration and wasting [7]. Various genetic mutations in one or more of the exons mainly cause out-of-frame translational reading, which prevents transcription and consequently causes complete loss of the protein [4,5].

Viltepso^TM^ binds to exon 53 of the dystrophin mRNA precursor, thereby masking the exon. It then allows restoration of the reading frame. The reading process proceeds, but skipping exon 53 and consequently producing a shorter, but still functional, dystrophin protein [4,5]. This outcome delays muscle deterioration and turns the overall condition into a Becker muscular dystrophy (BMD)-like condition, thus increasing the life expectancy of DMD patients. [4].

Viltepso^TM^ is the third PMO antisense oligonucleotide used to treat DMD by exon skipping. The first two were eteplirsin and golodirsin for exons 51 and 53, respectively [6,8]. Sharing the same structure as viltolarsen but with an additional four subunits and a triethylene glycol moiety at its 5′ end, golodirsin reached the market in 2019. Exon 51 skipping therapy is applicable to 14% of DMD patients, while exon 53 skipping is applicable to 10%. A multi-exon skipping approach is now under investigation and is expected to be beneficial for 45% of DMD patients [4].

Viltepso^TM^ is administered intravenously and has shown some adverse effects, including cough, nasopharyngitis, upper respiratory tract infection, vomiting, and diarrhea [5,9]. Developed in Japan by Nippon Shinyaku and in collaboration with the National Center of Neurology and Psychiatry (NCNP), it was first approved in Japan in March 2020 [5] and received authorization from the FDA on 12 August of the same year [10].

### 2.2. Lumasiran (Oxlumo^TM^)

**Figure 3:**
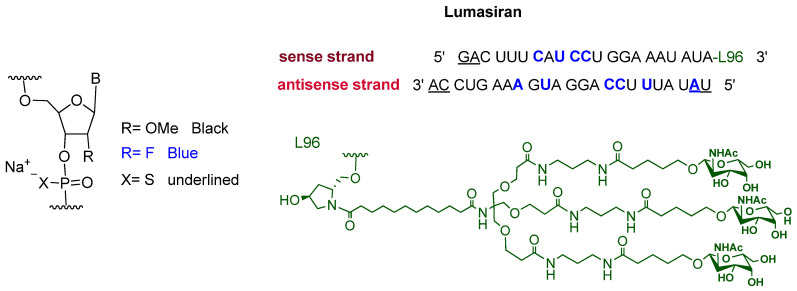
Chemical structure of lumasiran (Oxlumo^TM^).

Lumasiran is a double-stranded small interference RNA (siRNA). It consists of the sodium salt of a sense and an antisense strands containing some chemically modified units. Moreover, an N-acetyl galactosamine-bearing dendrimer is covalently attached to the 3′ terminus of the sense strand (Figure 3) [11]. After givosiran, which is used for the treatment of acute hepatic porphyria (AHP) and was approved in November 2019 [12], lumasiran is the second drug to exploit enhanced stabilization chemistry (ECT)-GalNAc conjugate technology [12,13].

Lumasiran is the first siRNA drug to be used for the treatment of primary hyperoxaluria type 1 (PH1), a hereditary disorder that results in the recurrent formation of kidney and bladder stones as a result of the deposition of endogenous oxalate as the poorly soluble calcium oxalate salt (CaOx) [14]. All previous treatments addressed mainly the symptoms rather than the primary cause [15]. The metabolism of hydroxyproline amino acid produces glyoxylate, which is further metabolized by certain hepatic enzymes to produce oxalate as final metabolite [16]. Oxalate is normally excreted in urine through the kidneys. Some genetic mutations cause a decrease in the production or activity of alanine-glyoxylate amino transferase (AGT), the enzyme responsible for breaking down glyoxylate. As a result, glyoxylate, and consequently the oxalate, accumulates [15,16,17]. Lumasiran (driven by the galactosamine moieties) targets hepatic cells and silences the gene encoding for glycolate oxidase (GO) enzyme, thereby halting its production. The depletion of this enzyme reduces the amounts of glyoxylate present and therefore the production of the oxalate [17].

The drug is administered subcutaneously and its main adverse effect is injection site reactions [11]. It was developed by Alnylam pharma and was approved by the FDA on 23 November 2020 [18].

## 3. Peptides

The last five years (2016–2020) have witnessed the authorization of 14 peptides, four being approved this year. These numbers reinforce the importance of peptides in the overall pharmaceutical market. Two of the class of 2020 are radiopharmaceuticals (^64^Cu and ^68^Ga), one a disulfide-containing peptide, and the last one the payload of an antibody drug conjugate (ADC). Table 3 shows the peptides approved by the FDA in the period 2016–2020.

### 3.1. Setmelanotide (Imcivree^TM^)

**Figure 4:**
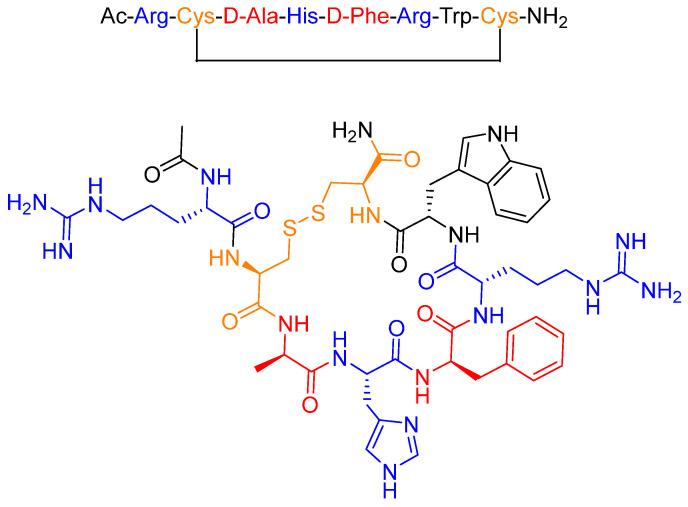
Chemical structure of setmelanotide (Imcivree^TM^).

Setmelanotide (formerly known as RM-493 or BIM-22493) is an eight-amino acid peptide that forms a cycle through a disulfide bridge between two Cys residues (Figure 4) [19]. The two D-amino acids (red) present in the sequence and its cyclic structure enhance resistance to proteolytic degradation, thereby increasing the half-life of the drug [20,21]. Furthermore, the positively charged amino acids (blue) are considered essential for ligand-receptor recognition and for activity [22].

Setmelanotide is prescribed for the treatment of rare genetic diseases of obesity caused by certain variants of the genes encoding for pro-opiomelanocortin (POMC), proprotein convertase subtilisin/kexin type 1 (PCSK1), or leptin receptor (LEPR) all of which cause a deficiency of these molecules [23]. Patients with such genetic alterations suffer from excessive or extreme hunger, severe early-onset obesity, and endocrine disorders [24,25].

Setmelanotide acts as a selective melanocortin-4 (MC4) receptor agonist and presents a 20-fold higher potency than the endogenous agonist α-MSH [19,26]. In the brain, MC4 receptors are involved in regulating hunger and satiety, in addition to energy expenditure [27]. Deficiency of POMC, PCSK1, and LEPR cause the MC4 receptor pathway to be insufficiently activated. Therefore, the binding of setmelanotide to the MC4 receptor reactivates the pathway, inducing a reduction of hunger that promotes weight loss due to decreased caloric intake and increased energy expenditure. After receiving setmelanotide, a noticeable reduction in body weight, as well as reversing hyperphagia, is observed within 45 to 61 weeks [22,28].

Setmelanotide shows high efficacy with fewer side effects, especially those related to hypertension and adverse cardiovascular effects, which were observed in the first generation MC4R agonists, such as LY2112688 [22,27,29].

It is administered subcutaneously and common adverse effects include skin hyperpigmentation, nausea, headache, diarrhea, abdominal pain, back pain, fatigue, vomiting, depression, upper respiratory tract infection, and spontaneous penile erection [23]. It also has several warnings and precautions, such as disturbance of sexual arousal, depression and suicidal ideation, skin pigmentation and darkening of pre-existing nevi, risk of serious adverse reactions in neonates, and low birth weight infants due to benzyl alcohol preservative [23]. It was developed by Rhythm Pharmaceuticals and was approved by the FDA on 25 November 2020 [30].

### 3.2. ^64^Cu -DOTATATE (Detectnet^TM^)

**Figure 5:**
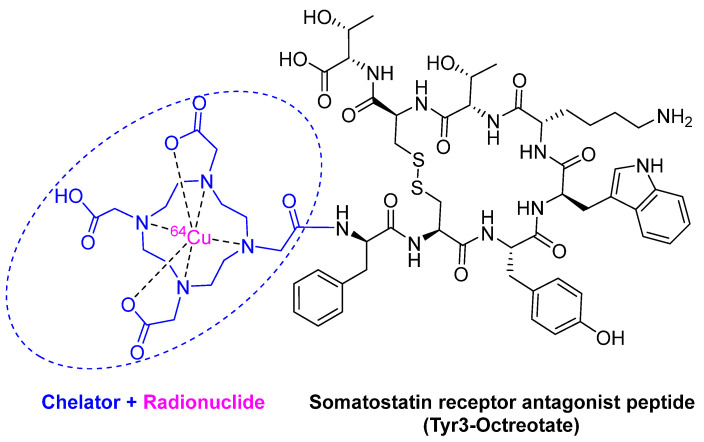
Chemical structure of ^64^Cu -DOTATATE (Detectnet^TM^).

^64^Cu -DOTATATE is composed of the radionuclide ^64^Cu (purple) chelated by DOTA (1,4,7,10-tetraazacyclododecane-1,4,7,10-tetraacetic acid) (blue), which is linked to the somatostatin analog octreotate peptide (black) (Figure 5). It is a radioactive diagnostic agent to be used with positron emission tomography (PET) for the localization of somatostatin receptor-positive neuroendocrine tumors (NETs) in adults [31]. Although ^64^Cu was previously used in nuclear medicine as ^64^Cu-diacetyl-bis(N4-methylthiosemicarbazone) (64Cu-ATSM) for PET imaging in head and neck cancer [32], and hypoxic myocardium [33], and in ^64^Cu-labeled anti-colorectal carcinoma mAbs (MAb 1A3) [34], ^64^Cu-DOTATATE is considered the first FDA-approved ^64^Cu-labeled radiopharmaceutical for Positron emission tomography–computed tomography (PET/CT) imaging.

Among the most important Cu radioisotopes, ^64^Cu is considered the most convenient for clinical applications since it has an intermediate half-life of 12.7 h and can decay via various routes, including electron capture, beta emission, and positron emission. Its low positron energy allows high quality and high-resolution images, plus the absence of abundant gamma emission (in contrast to other positron emitters), a type of emission that may also impair the imaging process [34]].

^64^Cu-DOTATATE outperforms ^68^Ga-DOTATOC, which was approved in 2019 [8]. This is explained by the fact that the radionuclide of ^68^Ga-DOTATOC has a shorter half-life (68 min), and its images are of lower resolution as a result of its higher positron energy [8]. A study by Johnbeck et al. showed that ^64^Cu-DOTATATE gave improved and greater detection of lesions than ^68^Ga-DOTATOC [35]. Furthermore, another study carried out by Pfeifer et al. also demonstrated a higher spatial resolution of the images taken by ^64^Cu- DOTATATE versus those by ^111^In-DTPA-octreotide and also reported greater detection of lesions by ^64^Cu-DOTATATE [36].

^64^Cu-DOTATATE is administered intravenously and has some adverse effects, including nausea, vomiting, and flushing [37]. It was developed by Radiomedix inc. and was approved by the FDA on 3 September 2020 [38].

### 3.3. ^68^Ga-PSMA-11

**Figure 6:**
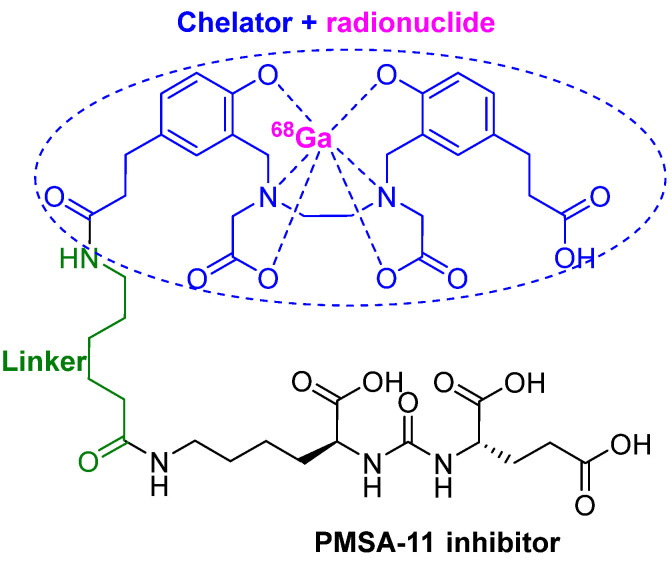
Chemical structure of ^68^Ga-PMSA-11.

^68^Ga-PSMA-11 or (^68^Ga gozetotide) consists of the lipophilic acyclic chelator HBED-CC (N,N′-bis [2-hydroxy-5-(carboxyethyl)benzyl] ethylenediamineN,N′- diacetic acid) (blue), which is coordinated to the radionuclide ^68^Ga (purple) and a urea-based peptidomimetic HO-Glu-NH-CO-NH-Lys-OH (black). Both moieties are linked by a 6-aminohexanoic acid (green) through the side chain (ε-NH_2_) of the Lys residue (Figure 6) [39,40].

^68^Ga-PSMA-11 is indicated for PET imaging of prostate-specific membrane antigen (PSMA)-positive lesions in men with prostate cancer [41]. It is the first PET drug to trace and detect prostate carcinoma relapses and metastases with high contrast by targeting the extracellular domain of the PSMA. It is a transmembrane glycoprotein expressed in the prostate, kidneys, small intestines, salivary glands, and brain [39,40]. However, it is overexpressed 100- to 1000-fold in prostate tumors compared to other tissues [40,42], thereby offering a specific target for diagnostic and therapeutic purposes.

The active binding site of PSMA combines a hydrophilic motif that interacts with the urea-based inhibitor Glu-NH-CO-NH-Lys- (its role being to drive the radionuclide to the cancer cells with high specificity), and a lipophilic pocket. Thus, the lipophilic nature of the HBED-CC chelator has a positive impact on the binding properties. Moreover, the chelator has shown high efficiency to complex with Ga(III) [40]. Compared to the corresponding DOTA-conjugate of the same pharmacophore, the HBED-CC chelator-conjugate demonstrated improved PET imaging performance as it showed reduced unspecific binding and considerably higher specificity, fast blood, and organ clearances, and low accumulation in liver [40].

Other conjugates, i.e., labeled antibodies, can also be used for targeting the cytoplasmic PMSA, such as ^111^In-capromab pendetide (ProstaScint^®^), which is used for scintigraphy. However, accessibility to the cytoplasmic PMSA compared to the extracellular protein is lower, especially for large molecules. Furthermore, the long half-life of ^111^In and, consequently, the long length of exposure to radiation and the prolonged circulation of the antibody in the body, which causes high background noise, make PET imaging with ^68^Ga more favorable than the scintigraphic method [42].

^68^Ga-PSMA-11 is administered intravenously and has some adverse effects, including nausea, diarrhea, and dizziness [41]. It was developed by the University of California (Los Angeles) and was approved by the FDA on 1 December 2020 [43].

### 3.4. Belantamab Mafodotin-Blmf (Blenrep^TM^)

**Figure 7:**
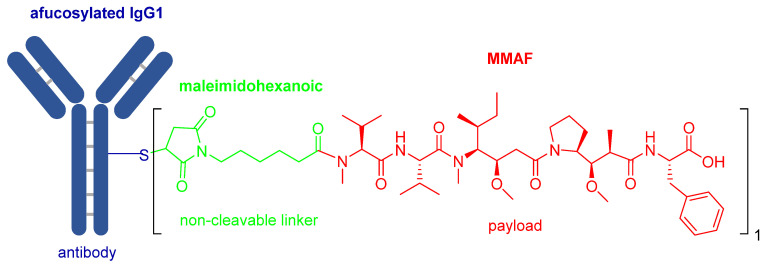
Chemical structure of belantamab mafodotin-blmf (Blenrep^TM^).

Belantamab mafodotin-blmf is the ninth ADC approved by the FDA. It is composed by afucosylated humanized immunoglobulin G1 monoclonal antibody (IgG1) (blue) and a peptide payload called monomethyl auristatin F (MMAF) (red) [44]. The two units are linked by the protease-resistant maleimidohexanoic linker (green) (Figure 7).

The afucosylated IgG1 antibody, which targets B-cell maturation antigen (BCMA) [45], is produced by recombinant DNA technology in a mammalian cell line (Chinese Hamster Ovary), whereas the payload-linker is prepared synthetically. Final attachment is by conjugation of the maleimide to the Cys residues on the antibody. To this end, a controlled reduction of various interchain disulfide bonds on the antibody must be achieved to prevent its denaturation. Usually, this step is done using the reducing agent tris-(2-carboxyethyl)-phosphine (TCEP), then the free thiol of such Cys residues react with maleimides under very mild conditions [46]. The conjugate obtained carries approximately four molecules of MMAF per antibody molecule.

**Figure 8:**
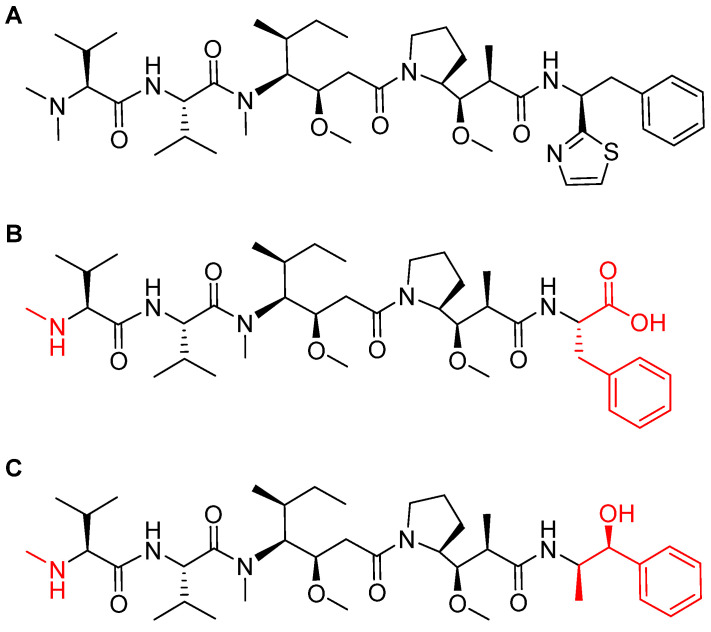
Chemical structure of: (**A**) natural dolastatin 10; (**B**) monomethyl auristatin F (MMAF); and (**C**) monomethyl auristatin E (MMAE). Differences from the natural molecule are shown in red [44].

MMAF is a synthetically modified analog of natural dolastatin 10 (Figure 8), which acts as an antimitotic agent, inhibiting cell division by blocking the polymerization of tubulin [45]. Due to the high cytotoxicity of both dolastatin 10 and its synthetic analogs, they cannot be administered directly but have to be delivered and deposited at the tumor site. The maleimidohexanoyl linker is considered non-cleavable because it is protease-resistant. Thus, catabolism of the mAb backbones is required to release the drug [47], in which a cysteine-adduct of the linker-MMAF derivative is released [44].

The ADCs approved in 2019 (Padcev and Polivy) used a monomethyl auristatin E (MMAE) analog as payload [8], which is more membrane-permeable than MMAF [48]. On the other hand, MMAF is more hydrophilic and this property decreases aggregation tendencies [48]. Most importantly, MMAF has lower bystander activities (drug release to neighboring antigen-negative cells or tissues) than MMAE [49]. Finally, MMAF shows improved pharmacokinetics and also a better therapeutic index [50] (Figure 8). Furthermore, the (Padcev and Polivy) were built using a cleavable linker. Of note, in contrast to ADCs with cleavable linkers, those with non-cleavable linkers have an extended plasma half-life as their bonds are not susceptible to lysosomal processes [51].

Belantamab mafodotin-blmf is prescribed for the treatment of adult patients with relapsed or refractory multiple myeloma who have received at least four prior therapies, including an anti-CD38 monoclonal antibody, a proteasome inhibitor, and an immunomodulatory agent [45]. Upon the binding of the mAb to the surface of the BCMA cell, belantamab mafodotin-blmf is internalized and MMAF is released via the proteolysis of the mAb component (as the linker is non-cleavable), ending with apoptosis [52].

Belantamab mafodotin-blmf is administered intravenously every three weeks [45,52]. It has various adverse effects: ≥20% are keratopathy (corneal epithelium change on eye exam), decreased visual acuity, nausea, blurred vision, pyrexia, infusion-related reactions, and fatigue; and ≥5% are decreased platelets, decreased lymphocytes, decreased hemoglobin, decreased neutrophils, increased creatinine, and increased gamma-glutamyl transferase.

Belantamab mafodotin-blmf was developed by GlaxoSmithKline and approved by the FDA on 5 August 2020 [53] and by the European Medicines Agency (EMA) on 25 August 2020 [52].

## 4. Conclusions

A total of 6 out of the 53 drugs approved by the FDA in 2020 belong to the TIDES category. The continued authorization of these chemical entities highlights their efficacy and safety profiles, and therefore the growing relevance of this family of drugs.

The peptide market has been strengthened over many years and this trend is expected to continue. During the preparation of this manuscript, it was disclosed that Plitidepsin^TM^ (aplidin), a peptide of marine origin approved a few years ago in Australia for multiple myeloma [54], shows potent preclinical efficacy against SARS-CoV-2 [55] and is expected to enter clinical phase III very shortly. Futhermore, on 22 January 2021, voclosporin (Lupkynis^TM^) was approved for the treatment of treat lupus nephritis [56]. Voclosporin is cyclosporine analogue.

Several newcomers to the oligonucleotide family, namely siRNAs and antisense oligonucleotides, are expected to join the pharmaceutical market arena shortly. Inclisiran, a siRNA for the treatment of hypercholesterolemia, was developed by Novartis under a license in collaboration with Alnylam Pharmaceuticals. It has been approved in Europe and is expected to receive authorization from the FDA soon [13,57,58]. Early members of the oligonucleotide drugs family demonstrated their efficacy in the treatment of some rare hereditary disorders. However, new promising candidates are now widening the applicability of this family for the treatment of more common diseases.

The applications of TIDES as drugs will undoubtedly continue to play an important role as drugs, as they are fuelled by the continuous research undertaken in both the academic and industrial setting.

## Figures and Tables

**Table 1:** Summary of the 2020 FDA peptides and oligonucleotides (TIDES) harvest.

#	Active Ingredient (Trade Name)	Type	Indication	Target	Route
1	Viltolarsen (Viltepso^TM^)	Antisense oligonucleotide	Duchenne’s muscular dystrophy (DMD)	DMD Exon 53	Intravenous
2	Lumasiran (Oxlumo^TM^)	Antisense oligonucleotide	Primary hyperoxaluria type 1 (PH1)	Hydroxyacid oxidase (glycolate oxidase) 1 (HOA1) mRNA	Subcutaneous
3	Setmelanotide (Imcivree^TM^)	Peptide	Chronic weight management (obesity)	Melanocortin-4 receptor (MC4R)	Subcutaneous
4	^64^Cu-DOTATATE (Detectnet^TM^)	Peptide	Scintigraphic imaging	Somatostatin receptor	Intravenous
5	^68^Ga-PSMA-11	Peptide	Diagnosis of recurrent prostate carcinoma	Prostate-specific membrane antigen (PSMA)	Intravenous
6	Belantamab mafodotin-blmf (Blenrep^TM^)	ADC ^a^ with peptide payload	Relapsed or refractory multiple myeloma	B-cell maturation antigen (BCMA)	Intravenous

^a^ ADC, antibody drug conjugate.

**Table 2:** FDA-approved oligonucleotides from 1998–2020.

#	Active Ingredient (Trade Name)	Company	Structure	Indication	Target	FDA Approval
1	Fomivirsen (Vitravene^TM^)	Ionis Pharma (Carlsbad, CA, USA) Novartis (Basel, Switzerland)	Antisense PS-ON ^a^	Cytomegalovirus retinitis	CMV UL123	August 1998
2	Pegaptanib (Macugen^TM^)	NeXstar Pharma (Boulder, CO, USA) Eyetech Pharma (Knolls, NJ, USA)	Polynucleotide aptamer	Neovascular age-related macular degeneration	VEGF-165	December 2004
3	Mipomersen (Kynamro^TM^)	Ionis Pharma (Carlsbad, CA, USA) Genzyme (Cambridge, MA, USA) Kastle Tx (Housten, TX, USA)	Antisense PS-ON ^a^	Homozygous familial hypercholesterolaemia	*APOB*	January 2013
4	Defibrotide (Defitelio^TM^)	Jazz Pharma (Dublin, Ireland)	Mixed single strands of DNA	Hepatic veno-occlusive disease	NA ^b^	March 2016
5	Eteplirsen (Exondys 51^TM^)	Sarepta Tx (De Berry, TX, USA)	Antisense PS-ON ^a^/(PMO ^c^)	Duchenne muscular dystrophy	*DMD* exon 51	September 2016
6	Nusinersen (Spinraza^TM^)	Ionis Pharma (Carlsbad, CA, USA) Biogen (Cambridge, MA, USA)	Antisense PS-ON ^a^/(PMO ^c^)	Spinal muscular atrophy	*SMN2* exon 7	December 2016
7	Patisiran (Onpattro^TM^)	Alnylam Pharma (Cambridge, MA, USA)	siRNA ^d^	Hereditary transthyretin amyloidosis polyneuropathy	*TTR*	August 2018
8	Inotersen (Tegsedi^TM^)	Ionis Pharma (Carlsbad, CA, USA) Akcea Pharma (Boston, MA, USA)	Antisense PS-ON ^a^	Hereditary transthyretin; Amyloidosis polyneuropathy	*TTR*	October 2018
9	Givosiran (Givlaari^TM^)	Alnylam Pharma (Cambridge, MA, USA)	siRNA ^d^	Acute hepatic porphyria	Aminolevulinate synthase 1 (ALAS1) mRNA ^e^	November 2019 (Enhanced)
10	Golodirsen (Vyondys 53^TM^)	Sarepta Tx (De Berry, TX, USA)	Antisense PMO ^c^	Duchenne muscular dystrophy	*DMD* exon 53	December 2019
11	Viltolarsen (Viltepso^TM^)	Nippon Shinyaku (Kisshoin, Minami-ku Kyoto) with (NCNP) ^f^ (Kodaira, Tokyo)	Antisense PMO ^c^	Duchenne muscular dystrophy	*DMD* exon 53	August 2020
12	Lumasiran (Oxlumo^TM^)	Alnylam Pharma (Cambridge, MA, USA)	siRNA ^d^	Primary hyperoxaluria type 1 (PH1)	HOA1 mRNA ^e^	November 2020

^a^ PS-ON: phosphorothioate oligonucleotide; ^b^ NA: not applicable, the mechanism of action is not well understood [3]; ^c^ PMO: phosphorodiamidate morpholino oligonucleotide; ^d^ siRNA: small interfering RNA; ^e^ mRNA: messenger RNA; ^f^ National Center of Neurology and Psychiatry.

**Table 3:** FDA-approved peptides between 2016 and 2020.

#	Active Ingredient (Trade Name)	Company	Structure	Indication	Target	FDA Approval
**Free Peptides**						
1	Lixisenatide (Adlyxin^TM^)	Sanofi-Aventis (Paris, France)	44 AAs	Diabetes type (II)	Glucagon-like peptide 1 receptor	July 2016
2	Plecanatide (Trulance^TM^)	Synergy Pharmaceuticals (New York, NY, USA)	16 AAs 2 disulfides	Chronic idiopathic constipation	Guanylate cyclase-C	January 2017
3	Etelcalcetide (Parsabiv^TM^)	KAI Pharmaceuticals (South of San Francisco, CA, USA) Amgen (Thousand Oaks, CA, USA)	7 AAs (all D) 1 disulfide intermolecular L-Cys	Secondary hyperparathyroidism in adult chronic kidney disease	Calcium-sensing receptor	February 2017
4	Abaloparatide (Tymlo^TM^)	Radius Health (Boston, MA, USA)	34 AAs	Anabolic agent	Parathyroid hormone 1 receptor	April 2017
5	Semaglutide (Ozempic^TM^)	Novo Nordisk (Måløv, Denmark)	31 AAs branched PEG-fatty acid	Diabetes type (II)	Glucagon-like peptide 1 receptor	December 2017
6	Macimorelin (Macrilen^TM^)	Aeterna Zentaris (Frankfurt, Germany)	3 residues pseudopeptide	Diagnosis of adult growth hormone deficiency	Growth hormone secretagogue receptor type 1	December 2017
7	Angiotensin II (Giapreza^TM^)	La Jolla Pharmaceutical (San Diego, CA, USA)	8 AAs	Septic shock, diabetes mellitus, and acute renal failure	Type-1 angiotensin II receptor	December 2017
8	Afamelanotide (Scenesse^TM^)	University of Arizona (Tucson, Arizona, USA) Clinuvel Inc. (Menlo Park, CA, USA)	13 AAs	Erythropoietic protoporphyria	Melanocortin 1 receptor	October 2019
9	Bremelanotide (Vyleesi^TM^)	Palatin Technology (East Windsor, NJ, USA) AMAG Pharmaceuticals (Waltham, MA, USA)	7AAs cyclic sidechain to tail	Hypoactive sexual desire disorder	Melanocortin receptors	June 2019
10	Setmelanotide (Imcivree^TM^)	Rhythm Pharmaceuticals (Boston, MA, USA)	8AAs Cyclic disulfide	Obesity	Melanocortin-4 receptor	November 2020
**Peptide-Chelator-radionuclide conjugates**						
11	[^177^Lu]-DOTA-TATE (Lutathera^TM^)	Advanced Accelerator Applications (Millburn, NJ, USA)	7AAs Cyclic disulfide	Gastroenteropancreatic neuroendocrine tumors	Somatostatin receptor	January 2018
12	[^68^Ga]-DOTATOC	University of Iowa Health Care (Iowa City, IA, USA)	A 7AAs Cyclic disulfide	PET imaging	Somatostatin receptor	August 2019
13	[^64^Cu]-DOTATATE (Detectnet^TM^)	Radiomedix Inc. (Housten, TX, USA)	A 7AAs Cyclic disulfide	PET imaging	Somatostatin receptor	September 2020
14	[^68^Ga]-PSMA-11	University of California (Oakland, CA, USA)	Peptidomemitic 2AAs urea linked	Diagnosis of recurrent prostate carcinoma by PET	Prostate-specific membrane antigen	December 2020
**Peptides in ADC’s**						
15	Enfortumab vedotin-ejfv (Padcev^TM^)	Astellas Pharma (Northbrook, IL, USA)	5 residues with γ-AA	Urothelial cancers	Nectin-4 receptor	December 2019
16	Polatuzumab vedotin-piiq (Polivy^TM^)	Roche (South of San Francisco, CA, USA)	5 residues with γ-AA	Refractory diffuse large B-cell lymphoma	CD79b receptor expressed in mature Bcells	June 2019
17	Belantamab mafodotin-blmf (Blenrep^TM^)	GlaxoSmithKline (Brentford, UK)	5 residues with γ-AA	Relapsed or refractory multiple myeloma	B-cell maturation antigen	August 2020
